# Transcriptomic Changes in the Frontal Cortex of Juvenile Pigs with Diet-Induced Metabolic Dysfunction-Associated Liver Disease

**DOI:** 10.3390/biomedicines13071567

**Published:** 2025-06-26

**Authors:** Kyle Mahon, Mohammed Abo-Ismail, Emily Auten, Rodrigo Manjarin, Magdalena Maj

**Affiliations:** 1 Biological Sciences Department, California Polytechnic State University, San Luis Obispo, CA 93407, USA; kmahon@calpoly.edu; 2 Animal Science Department, California Polytechnic State University, San Luis Obispo, CA 93407, USA; maboisma@calpoly.edu (M.A.-I.); rmanjari@calpoly.edu (R.M.); 3 Kinesiology Department, California Polytechnic State University, San Luis Obispo, CA 93407, USA; eauten13@gmail.com

**Keywords:** neurodegeneration, pediatric MASLD, transcriptomics, brain, Iberian pig, Wnt pathway, mitochondria, extracellular matrix

## Abstract

**Background/Objectives**: Neurodegenerative disorders have a complex multifactorial pathogenesis that develop decades before the initial symptoms occur. One of the crucial factors in the development of neurodegenerative disorders is an unbalanced diet. A pediatric animal model of diet-induced metabolic dysfunction-associated steatotic liver disease (MASLD) was established by feeding juvenile Iberian pigs a diet high in fat and fructose for 10 weeks. The aim of this study was to investigate the initial molecular imbalances in the frontal cortex (FC) of diet-induced juvenile MASLD pig model and determine whether these changes are associated with neuronal loss. **Methods**: Eighteen 15-day-old Iberian pigs were randomly assigned to either a standard diet (SD) or a Western diet (WD) for 10 weeks. A short-term recognition memory test and animal activity was recorded during the study. Animals were euthanized in week 10, and the FC and hippocampus (HIP) tissue samples were collected for immunohistochemistry and transcriptomics analyses. **Results**: WD-fed pigs developed MASLD. There were no significant differences in animals’ activity or recognition memory between WD and SD. To identify and quantify mature neurons, NeuN immunostaining intensity was measured, which was significantly lower in the FC of WD than SD (*p* ≤ 0.05), but it did not change in HIP (*p* ≥ 0.05). The Wnt/β-catenin pathway, which promotes neuronal survival and neurogenesis, was downregulated in FC of WD-fed pigs (*p* ≤ 0.05). Similarly, cytoskeleton organization and extracellular matrix biological processes were downregulated in FC of WD-fed pigs (*p* ≤ 0.05), whereas the mitochondrial respiratory chain complex and mitochondrion increased in FC of WD compared with SD (*p* ≤ 0.01). There were several other significantly modulated pathways including signal transduction, cell migration, axon guidance, and calcium ion binding. **Conclusions**: The high-fructose, high-fat diet led to neuronal loss in the frontal cortex of MASLD pigs and dysregulated gene expression of the Wnt/β-catenin signaling pathway, cytoskeleton organization, extracellular matrix, and mitochondrial respiratory chain—all pathways that are found deregulated in neurodegnerative diseases.

## 1. Introduction

The prevalence of neurodegenerative disorders such as Alzheimer’s disease (AD) and Parkinson’s disease (PD) continues to increase globally due to an aging and growing society, currently affecting ~15% of the worldwide population [1,2]. Neurodegenerative disorders (NDs) are characterized by progressive damage to the nervous system which often starts developing decades before the symptoms appear, making NDs very difficult to prevent or treat. It is believed that the initial molecular imbalances contribute to neuroinflammation and loss of structure and function of neurons, gradually leading to neuronal loss [3,4]. Although NDs have a complex multifactorial pathogenesis, nutrition plays an essential role in the development and progression of these diseases [5,6]. Recent epidemiological and animal studies linked diets high in sugar and/or fat to an increased risk of NDs [7,8,9,10]. In this line, our team, by feeding a high-fructose high-fat diet, has recently established a large animal model of diet-induced neurodegeneration [11]. After only 10 weeks, juvenile Iberian pigs developed metabolic dysfunction-associated steatotic liver disease (MASLD) (previously named NAFLD), gut dysbiosis, colonic hyperplasia, and brain injury [11,12], representing the earliest age of diet-induced MASLD and neurodegeneration. This large non-ruminant pediatric animal model allows us to investigate the mechanisms by which early-life nutritional insults may impact the degenerative processes in the brain. This is especially important given that the prevalence of pediatric MASLD increased drastically in the last decade, currently diagnosed in ~10% of children and adolescents in the general population and up to 50% in children and adolescents with obesity [13]. Pediatric MASLD, linked to overnutrition, is characterized by hepatic steatosis, liver inflammation, and hepatocytes injury [14,15] and represents the main cause of pediatric chronic liver disease [16,17]. With proper dietary intervention and an increase in physical activity MASLD can revert in children [13,18,19]; however, it can also progress to metabolic dysfunction-associated steatohepatitis (MASH) and liver cirrhosis. Interestingly, there are several molecular and cytogenetic factors that play a role in the development and progression of MASLD, such as single nucleotide polymorphisms in *PNPLA3*, *TM6SF2*, *GCKR*, *MBOAT7*, and *HSD17B13* genes, all of which play a role in lipid metabolism [20].

The liver–brain axis sets an interconnection between two organs, in which liver pathologies may promote brain injury. In this respect, MASLD was linked to smaller total cerebral brain volumes in adults [21,22], while another study showed an association between MASLD and lower cognitive performance using a serial digital learning test, which evaluates learning, recall, and concentration [23]. Data from rodent models also support that liver disease induces or accelerates AD hallmarks, such as neuroinflammation, neuronal apoptosis, and increased amyloid-β plaque load [24,25,26]. However, the molecular mechanisms by which diet-induced liver pathology leads to neurodegeneration remain elusive.

Given many similarities between the human and swine anatomy and physiology of the liver and brain, pigs have been recognized as an excellent translational model to study MASLD and brain injury [27,28,29]. Neonate pigs and human infants both have gyrated brains, similar distributions of white and gray matter, myelination, electrical activity, and a comparable postnatal brain growth with no significant changes in the number of neurons between neonates and adult animals [29]. In addition, pigs can be tested in learning and memory tasks at an early age [30], and they develop markers of neurodegeneration, such as amyloid-β plaques and hyperphosphorylated-TAU [31]. Importantly, in both humans and pigs, the brain undergoes an accelerated postnatal development, and this early phase of life is recognized as a period of increased vulnerability to injury by environmental insults, such as diet.

Therefore, the objective of the current study was to use a transcriptomic discovery approach to investigate molecular mechanisms leading to neurodegeneration in a diet-induced pig model of juvenile MASLD. We hypothesized that a hypercaloric diet enriched in fat and fructose will lead to gene expression changes in the frontal cortex of juvenile MASLD Iberian pigs contributing to dysregulation of signaling pathways essential for neuronal survival.

## 2. Materials and Methods

### 2.1. Animals and Experimental Design

The experiments were conducted with approval by the Institutional Animal Care and Use Committee of California Polytechnic State University (#1611) and followed the Care and Use of Laboratory Animals guidelines issued by the National Research Council Guide. Eighteen male (M) and female (F) juvenile Iberian Pigs (15 ± 3 d of age) were housed in pairs and assigned randomly for 10 weeks to either a standard diet (SD, n = 8 pigs, 6M/2F), or a Western diet (WD, n = 10, 6M/4F) high in fructose and fat to induce MASLD. The composition of the diets and the daily nutrient and metabolizable energy are presented in Table 1 and Table 2, respectively. Pigs were fed 45 mL/kg body weight (BW) of their assigned diet every 6 h to match the physiological volume of milk consumed during the lactation period. The BW was recorded every 3 days, and subsequently the food intake was appropriately adjusted. All animals consumed an entire meal within 60 min and had free access to water. At week 10 and after 8 h post feeding, pigs were euthanized as described in previous studies [12,32]. None of the animals were excluded from the analyses. Brain weight was recorded promptly after euthanasia, and tissues from the frontal cortex (FC) and hippocampus (HIP) were collected and washed for 5 s in ice-cold saline solution. Then, the tissues were either frozen in liquid nitrogen or set in plastic cassettes (Tissue-Tek Cryomold Standard; Sakura, Torrance, CA, USA) that were covered using optimum cutting temperature compound (OCT) (Cat. No. 4583, Tissue-Tek O.C.T; Sakura, Torrance, CA, USA) and gradually frozen in liquid nitrogen-cooled 2-methylbutane (Cat. No. M0167, TCI, Portland, OR, USA). Tissues were stored at −80 °C until further processing.

### 2.2. Pen Activity and Novel Object Recognition Test

Physical activity in the pen was recorded and quantified as described in a previous study [11]. Briefly, cameras mounted from the ceiling recorded animal activity between d 16 and d 70 from 8:30 AM (1 h post feeding) to 12:30 PM. Animals’ activity was scored by two independent experimenters blinded to the diet using Behavioral Observation Research Interactive Software (BORIS; version 7.9) [33]. Experimenters used the ethogram detailed in a previous neurobehavioral study with juvenile Iberian pigs [34]. The activities of animals in each pen were scored individually and expressed as a mean percentage of each day in which the animals were active.

To assess recognition memory, the novel object recognition test was performed as described before [30]. The test was performed once a week between d 35 and d 70 of the study, as previous findings indicate 5-week-old domestic pigs can remember objects for up to 6 days [35]. In brief, after 1 h of the morning feeding, the test would begin by attaching two identical sample objects to the gates of each pen using zip-ties to keep the objects in place. In the sample phase, the pigs were given 10 min to investigate the objects. After 1 h, a new object of the same color but a novel shape was affixed to the pen gates along with one sample object. During this test phase, the pigs were given another 10 min to explore the objects. The video recordings of the two phases were scored by two independent experimenters blinded to the diet using BORIS software [33]. The scoring was based on the previously detailed ethogram [34], with data being presented as recognition index (RI; calculated by dividing time spent investigating novel object by time spent investigating both objects).

### 2.3. Immunofluorescence Analysis

To assess the number of mature neurons, immunofluorescence staining against neuronal nuclei (NeuN) was performed on the FC and HIP tissue as previously described [11]. Stains were incubated overnight with a primary antibody against NeuN (Cat. No. MAB377, NeuN, MilliporeSigma, Burlington, MA, USA). A blinded experimenter captured 10–15 images per tissue sample using the 40× objective of a FluoView 500 Confocal Laser Scanning Microscope (Olympus; Center Valley, PA, USA). ImageJ software version 1.54h [36] was used to convert the images to a Z-projection allowing for quantification of the average staining intensity which is reported as percentage of total area.

### 2.4. Transcriptomics Analysis

The effect of the WD-induced brain injury was further investigated through transcriptome-wide RNA profiling of the FC tissue. We performed transcriptomics analysis on the FC because it is a brain region in which neurons are among the first to deteriorate in NDs such as Alzheimer’s disease. Five FC tissue samples from each WD- and SD-fed animals were sent to GENEWIZ, LLC. (South Plainfield, NJ, USA) for RNA isolation, library preparation, and sequencing as described previously [34]. Briefly, the RNeasy Plus Mini Kit (Cat. No. 74134, Qiagen, Hilden, Germany) was used to extract the total RNA, which was quantified by a Qubit 2.0 Fluorometer (Life Technologies, Carlsbad, CA, USA), and assessed for integrity using the TapeStation 4200 automated electrophoresis tool (Agilent Technologies, Palo Alto, CA, USA). Messenger RNA libraries were prepared in accordance with the manufacturer’s instructions (Cat. No. E7770, NEB, Ipswich, MA, USA) for proper use of the NEBNext Ultra RNA Library Prep Kit for Illumina. Sequencing of the RNA libraries was conducted using a 2 × 150 bp Paired-End configuration of the Illumina HiSeq 4000 system on 2 flow cell lanes. Following this procedure, the resulting sequence quality was evaluated using FastQC software version 0.12.1 [37] and a read-count table was produced using the feature Counts package in Subread software version 2.0.1 [38].

### 2.5. Statistical Methods

Data on brain weight, number of mature neurons, and RI were analyzed using a univariate mixed linear model by procedure mixed implemented in SAS 9.4 (PROC MIXED, SAS Institute Inc., Cary, NC, USA). The model included the diet as a fixed-effect pen nested in diet as a random effect, and time as repeated measure. The significance was established at *p* ≤ 0.05. The edgeR-Bioconductor package in R software version 4.3.2 was used to perform the statistical analyses of transcriptomic data [39]. Differentially expressed genes (DEGs) between the SD and WD treatments were identified using a generalized linear model assuming a negative binomial distribution of gene counts at a 5% false discovery rate (FDR). Then, functional enrichment analyses were conducted on DEGs using the Database for Annotation, Visualization and Integrated Discovery (DAVID) software version 6.8 [40] to identify gene ontology (GO) terms and biological pathways enriched in the detected DEGs. The association between GO terms and relevant DEGs was illustrated in heatmaps created with ClustVis software (BETA) version 0.12.0 [41].

## 3. Results

### 3.1. Western Diet-Fed Pigs Had Increased Frontal Cortex Neuronal Loss Without Changes in Brain Weight, Physical Activity, or Cognitive Function

The goal of this study was to examine the effects of a Western diet (WD) on brain health in a juvenile pig model of MASLD. The MASLD phenotype observed in the juvenile Iberian pigs used in this study, evaluated by biochemistry, histology, and metabolomics in the liver, blood, and gut, has been detailed in our previous report by Maj et al. 2023 [42]. Briefly, steatotic grade (*p* ≤ 0.001), necrosis (*p* ≤ 0.001), hepatocellular proliferation (*p* ≤ 0.01), and composite lesion score (*p* ≤ 0.001) in liver tissues were higher in WD compared with SD (Table 2). Similarly, serum biochemistry showed an increase in alanine transaminase (*p* ≤ 0.05), aspartate transaminase (*p* ≤ 0.001), alkaline phosphatase (*p* ≤ 0.001), gamma glutamyl transferase (*p* ≤ 0.05), and lactate dehydrogenase (*p* ≤ 0.01) in WD compared with SD (Table 3).

**Figure 1:**
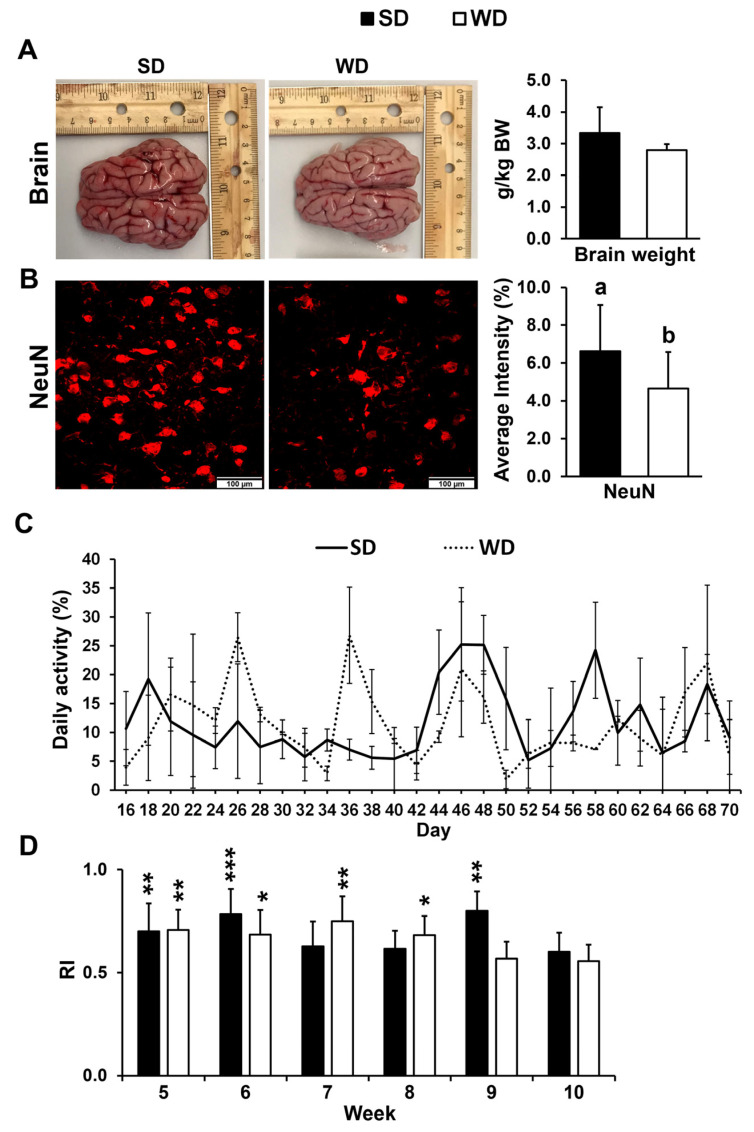
(**A**) Representative brain images and average brain mass per kg body weight (BW) from the pigs fed the standard (SD) and Western (WD) diets; images were taken directly after euthanasia on day 70 of the study. (**B**) Neuronal nuclei (NeuN) immunofluorescent staining of the frontal cortex (FC) tissue (left—representative examples) and quantification reported as an average staining intensity (%) in the SD and WD-fed animals. Values are means ± standard deviation (sd). Letters represent significant differences: ^ab^ *p* ≤ 0.05. (**C**) Animal activity in pen, measured every other day from 8:30 AM to 12:30 PM between days 16 and 70 of the study. Values are reported as means ± sd. (**D**) Novel object recognition test was performed once a week starting in week 5 of the study, and the results are presented as a recognition index (RI). Significant *p* values for 1-tailed t tests and *p* values adjusted for multiple testing with Tukey’s post hoc tests, are expressed as * *p* ≤ 0.05, ** *p* ≤ 0.01, and *** *p* ≤ 0.001. Values are means ± sd.

Relative brain weight did not show a significant difference between two nutritional treatments (Figure 1A). The number of mature neurons was significantly lower in the FC of WD-fed pigs (*p* ≤ 0.05) (Figure 1B) but not in the HIP (*p* > 0.05). No significant differences in daily activity were found between the two treatment groups over the course of the 10-week study (Figure 1C). The RI in the novel object recognition test was >0.5 for SD and WD in week 5 through week 10. However, there were no differences in recognition index (RI) between the two treatments at any given time (*p* ≤ 0.05) (Figure 1D).

### 3.2. Western Diet-Fed Pigs Had Dysregulated Cortical Genes Associated with the Wnt/β-Catenin Signaling Pathway, Organization of the Cytoskeleton and Extracellular Matrix, and Mitochondrial Function

The effect of the WD-induced brain injury was further investigated through transcriptome-wide RNA profiling of the FC tissue. Given that we observed a neuronal loss only in the FC tissue but not in the HIP, we did not perform transcriptome analysis of the hippocampal tissue. An average of about 15,000 individual transcripts per animal were fitted into a generalized linear model analysis. The current study detected 176 differentially expressed genes (DEGs) between WD and SD treatments with 125 downregulated and 51 upregulated genes. Thus, the in silico functional enrichment analyses were conducted on these 176 identified DEGs for *biological process*, *cellular component*, and *molecular function*. The GO terms with statistically significant absolute *p*-values (*p* ≤ 0.05) were listed in Table 1.

The major categories in the *biological process* ontology, which are the larger biological processes carried out by the action of multiple molecular activities, were mitochondrial respiratory chain complex I and mitochondrial electron transport, cell migration, canonical Wnt signaling pathway, signal transduction, cytoskeleton, and ECM organization (Table 4).

The major categories in the *cellular component* ontology, which represent the cellular location where a molecular function occurs, were linked to mitochondria (mitochondrial respiratory chain complex I, mitochondrial inner membrane, mitochondrial nucleoid), cell surface (basement membrane, focal adhesion, cell junction, adherens junction), extracellular matrix, and Wnt/beta-catenin pathway (β-catenin TCF complex) (Table 4).

Lastly, the major categories in the *molecular function* ontology, which describe molecular-level activities performed by gene products, include mitochondria (electron carrier activity, oxidoreductase activity), Wnt pathway, extracellular matrix, and ion channel activity (Table 4).

**Figure 2:**
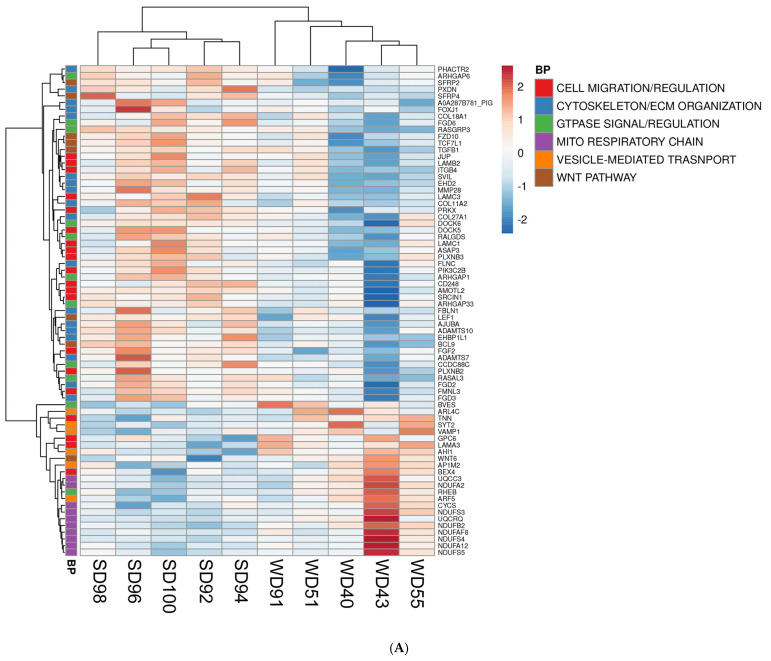

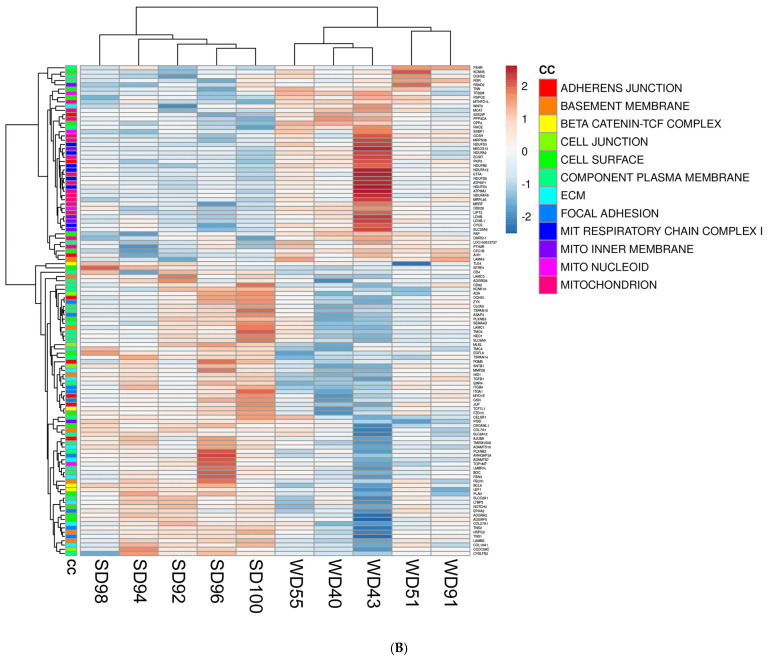

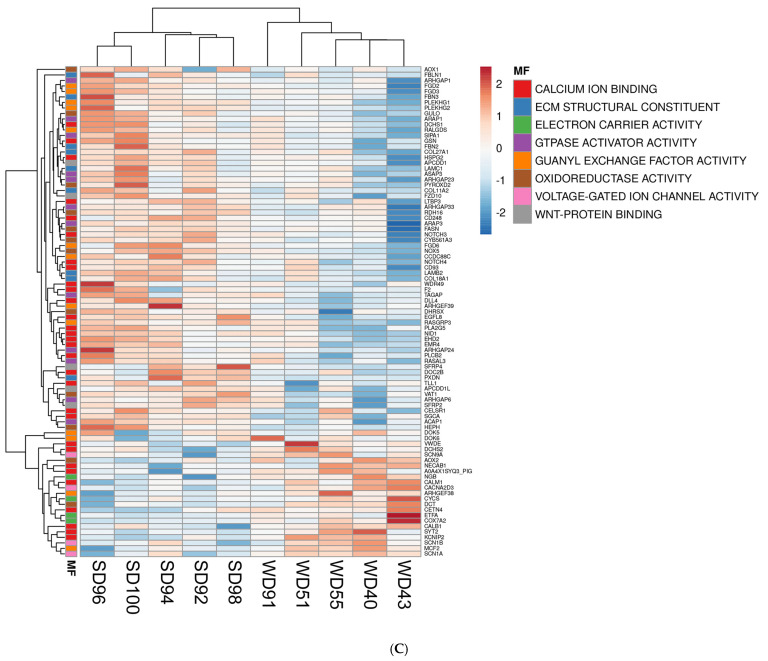
Heat maps of differentially expressed genes (DEGs) (5% FDR) in the frontal cortex of the SD and WD animals on day 70 of the study: (**A**) *biological processes*, (**B**) *cellular components*, and (**C**) *molecular functions*. Individual pigs are represented in each column, and rows are expressed as each gene’s log-transformed read counts-per-million. The blue color indicates the row minimum, and red represents the row maximum. DEGs, differently expressed genes; FDR, false discovery rate; WD, Western diet; SD, standard diet.

Furthermore, the association between GO terms and relevant DEGs was displayed as heatmaps (Figure 2). Because many GO classes are overlapping or redundant, below we present major transcriptomics results by integrating individual components of a major category from all three GO areas (*biological process*, *cellular component*, and *molecular function*) into a unified pathway.

The FC of WD-fed pigs showed a downregulation of multiple Wnt/β-catenin signaling pathway genes as compared to SD animals. The Wnt/β-catenin pathway genes are involved in cell fate determination, polarity, and neural patterning, including Secreted Frizzled Related Proteins 2 and 4 (*SFRP2*, *SFRP4*), Frizzled Class Receptor 10 (*FZD10*), Transcription Factor 7 Like 1 (*TCF7L1*), and Transforming Growth Factor Beta 1 (*TGFB1*) (Figure 2A–C). Conversely, the FC of WD-fed pigs displayed an upregulation of Wnt Family Member 6 (*WNT6*), a ligand for the Wnt signaling pathway (Figure 2A).

Transcriptomic analysis revealed consistent upregulation of genes involved in mitochondrial function related to the construction and function of the first enzyme complex in the electron transport chain (ETC) of the FC of WD pigs. These genes include NADH:Ubiquinone Oxidoreductase Subunit A2, A12, B2, S3, S4, and S5 (*NDUFA2*, *NDUFA12*, *NDUFB2*, *NDUFS3*, *NDUFS4*, and *NDUFS5*) (Figure 2A,B). The frontal cortex of WD animals also showed an upregulated expression of genes associated with interconversion of NADH and NAD+ and the transport of electrons within the ETC, such as Lactate Dehydrogenase B (*LDBH*) and *Cytochrome C*, respectively (Figure 2A,B).

Furthermore, the FC of WD animals displayed a downregulation of genes associated with the cytoskeleton and ECM organization (Figure 2A), such as Phosphatase and Actin Regulator 2 (*PHACTR2*), Peroxidasin (*PXDN*), Collagen Type XVIII Alpha 1 Chain (*COL18A1*), Supervillin (*SVIL*), EH Domain Containing 2 (*EHD2*), Filamin C (*FLNC*), EH Domain Binding Protein 1 Like 1 (*EHBP1L1*), *FYVE* (gene named after the first four proteins it was found in: Fab1p, YOTB, Vac1p, and EEA1), Rho Guanine Nucleotide Exchange Factor (*RhoGEF*), and PH Domain Containing 2 and 3 (*FGD2*, *FGD3*).

Lastly, the FC of WD pigs had a downregulation of calcium-ion-bonding-related gene levels (Figure 2C), which are involved in intercellular signaling and cell-fate determination and include Notch Receptors 3 and 4 (*NOTCH 3*, *NOTCH 4*), Delta Like Canonical Notch Ligand 4 (*DLL4*), EGF Like Domain Multiple 8 (*EGFL8*), and Nidogen 1 (*NID1*). Calcium dependent genes which play a role in cell–cell adhesion, signal transduction, and vesicle motility were also dysregulated in the FC of WD animals compared to SD but were not consistently upregulated or downregulated (Figure 2C).

## 4. Discussion

The objective of this study was to utilize a transcriptomic approach to detect the genes and molecular pathways contributing to neuronal loss in a diet-induced pediatric pig model of MASLD. Of note, gene expression does not always correlate with protein expression or activity, and, therefore, the transcriptomic results from this study need to be further verified by protein expression analysis. Juvenile Iberian pigs, which are known to be an excellent animal model to study metabolic pathologies, were fed either a Western diet high in fat and fructose or a standard diet for 10 weeks. Our results showed neuronal loss and dysregulated gene expression of brain injury markers in the frontal cortex of WD-fed pigs. Identified DEGs in the FC of WD animals were involved in multiple cellular pathways: the canonical Wnt/beta-catenin signaling pathway, cytoskeleton/ECM organization, mitochondrial respiratory chain, signal transduction, cell surface, and ion channel activity, all of which are known to be dysregulated in NDs. Nonetheless, the physical activity of animals and cognitive function did not differ between the two treatment groups. Our results suggest that the observed molecular and cellular differences indicative of neurological decline precede cognitive impairment and shed light on the etiology of diet-induced pathological trajectories in the brain. This goes in line with clinical data in which the loss of structure and function of neurons occurs prior to the symptoms of NDs [3,4].

**Figure 3:**
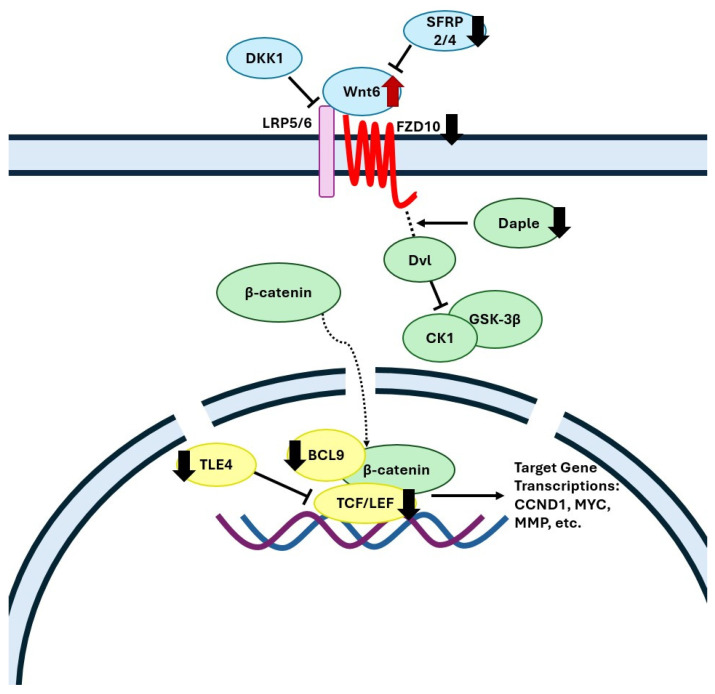
Mechanistic illustration of the frontal cortex genes in canonical Wnt signaling pathway altered by diet. Red and black arrows represent upregulated and downregulated genes in WD-fed pigs compared with SD, respectively. FZD, frizzled class membrane receptors; LRP5/6, low-density lipoprotein receptors; Dvl, dishevelled protein; CCDC88C, Daple protein; CK1, casein kinase 1; GSK-3β, glycogen synthase kinase-3 beta; TCF/LEF, T-cell factor/lymphoid enhancer factor; Cyclin D1 (CCND1), c-Myc (MYC), and matrix metallopeptidase (MMP). Created with Microsoft PowerPoint.

The current transcriptomics analysis revealed that gene ontology terms for the Wnt/β-catenin signaling pathway were downregulated in the FC of WD-fed animals, which is in agreement with clinical data showing an overall downregulation of this pathway in the FC of Alzheimer’s disease patients [43,44]. In the brain, the canonical Wnt/β-catenin signaling pathway plays a critical role in neuronal survival, neurogenesis, axon guidance, synaptic plasticity, and neuroprotection [45,46,47,48]. In the FC of WD animals, we found a decreased expression level of a Wnt receptor Frizzled-10 (*FZD10*) (Figure 3, which has been previously shown to be downregulated in the brain of aging mice, resulting in diminished Wnt signaling [49]. Gene expression of the Daple protein (*CCDC88C*), which assists in the release of Dvl from the frizzled receptor, and of Adhesion G Protein-Coupled Receptor A2 protein (*ADGRA2*), which forms a receptor complex recruiting Dvl upon binding with the Wnt7 ligand [50], were decreased in the WD-fed pigs (Figure 3). Wnt7 signaling is involved in proper neurovascular function, forming a network of blood vessels that encompasses the blood–brain barrier (BBB) [51]; hence, its dysregulation may contribute to a lack of BBB integrity. Furthermore, WD-fed animals displayed a downregulation of nuclear β-catenin transcription agonists *LEF1*, *TCF7L1*, and *BCL9* indicating diminished Wnt signaling. Importantly, patients with AD show the same expression patterns of *LEF1* and *TCF7L1* as the WD-fed pigs [52,53,54].

The mitochondrial respiratory chain biological process and the mitochondrial structure genes were mostly upregulated in the FC of WD-fed animals. Mitochondrial dysfunction leading to altered energy homeostasis has been identified as one of the main cellular defects underlying the pathogenesis of diverse NDs [55,56,57]. While advanced stages of neurodegeneration are characterized by mitochondrial dysfunction and a decrease in mitochondrial gene expression and mitochondrial enzymatic activity [58], it is believed that initial stages of metabolic disorders and NDs are often characterized by mitochondria-associated gene upregulation [59]. In this respect, we found multiple DEGs related to mitochondrial electron transport, electron carrier activity, and mitochondrial structure to be upregulated in the FC of WD-fed animals. The FC of WD animals showed an increased gene expression of mitochondrial subunit complexes and electron transporters, such as cytochrome c. Complex I consists of multiple protein subunits, including *NDUFB2*, *NDUFA2*, *NDUFS3*, *NDUFAF8*, *NDUFS4*, *NDUFA12*, and *NDUFS5*, which were all upregulated in WD animals (Figure 2A,B). Similarly, *Complex I* subunits have been previously found to be upregulated in the brains of porcine obesity models [60]. The gene expression of subunit *NDUFB2*, was found decreased in the brains of PD patients but increased in the adult aging brain [61]. Mitochondria are also involved in the initiation of apoptosis [62]; *CYCS* gene encoding cytochrome c was upregulated in the FC of WD pigs, and this increase was previously associated with elevated rates of cell death [62], while BCL2 protein, which prevents cytochrome c release [63], has been found reduced in WD-fed pigs and in the transcriptomes of patients consuming a high fat, ketogenic diet [64].

We also found that gene ontology terms for the cytoskeleton/extracellular matrix organization were mostly downregulated in the FC of WD fed animals. The ECM has been recently recognized to be involved in the process of neurodegeneration by contributing to synaptic and neuronal loss [65,66]. We found in the FC of WD-fed pigs that one of the key components of the ECM, *actin filaments* and their assisting organization proteins, such as *gelsolin*, *supervillin*, and *filamin C*, were all downregulated (Figure 2A–C). Importantly, actin cytoskeleton is essential for neurite outgrowth, axon guidance, dendritic spine morphology, and synaptic plasticity [67]. Furthermore, genes linked to ECM function were also downregulated in the FC of WD pigs, and this is consistent with ECM expression patterns found in AD patients [68,69].

Another important aspect for brain function is adhesion between neurons and the ECM, which is crucial for neuronal structure and synaptic plasticity, while disruptions in cell–ECM interactions can contribute to NDs [66,70]. The FC of WD pigs had decreased expression level of genes encoding proteins facilitating cell–cell and cell–matrix adhesion. For example, laminin subunit gamma 1 (*LAMC1*), which encodes a laminin subunit necessary for basement membrane formation, was downregulated, a pattern also found in aging human brains [71]. Genes encoding dachsous cadherin-related 1 and 2 (*DCHS1*, *DCHS2*), the two forms of cadherin domains critical for synapse formation, were dysregulated in the FC of WD animals. Decreased level of *DCHS1*, as found in the FC of WD-fed pigs, has been shown to negatively impact the differentiation of neural stem cells [72], while an upregulation of *DCHS2* expression, as detected in the FC of WD-fed pigs, has been associated with increased levels of amyloid-beta, a key hallmark of AD [73].

Interestingly, dietary patterns can greatly influence synaptic plasticity throughout life [74,75,76]. For example, diets enriched in saturated fatty acids significantly decreased synaptic plasticity in rodents [77]. The development of plasticity is attributed to numerous pathways, including the Notch signaling pathway, which additionally has roles in neurogenesis, neuronal growth, and differentiation, while impairment of Notch signaling has been associated with neuronal death, cognitive decline, and AD [78]. In this study, in the FC of WD-fed pigs, we found downregulation of several Notch signaling genes, such as the Notch receptors *NOTCH3* and *NOTCH4,* and the Notch ligand Delta Like Canonical Notch Ligand 4 (*DLL4*). Activation of NOTCH3 and NOTCH4 receptors initiate the transcription of genes controlling the function and integrity of the BBB [79], and a decline in Notch signaling has been associated with age-related human brain vascular deficiencies, BBB disruption, and NDs [80,81,82]. We also found that the FC of WD-fed pigs displayed a downregulation of genes involved in neuron differentiation and morphology, including dendritic spine formation, both essential structural factors in neuroplasticity. For example, WD-fed pigs showed a decreased level of DS Cell Adhesion Molecule Like 1 (*DSCAML1*) gene, which has a role in neuron differentiation and development [83]. Likewise, the SRC Kinase Signaling Inhibitor 1 (*SRCIN1*) gene, which has a regulatory role in the morphogenesis of dendritic spines, was decreased in the FC of WD-fed pigs, similarly as found in hippocampus of AD patients [84].

Lastly, it has been previously shown that dietary patterns may influence the level of neurotransmitters and the expression level of SNARE (soluble *N*-ethylmaleimide sensitive factor attachment protein receptor) proteins, both factors being critical for the process of neurotransmission [85,86]. In the current study, the FC of WD-fed animals displayed an upregulation of vesicle associated membrane protein 1 (*VAMP1*) gene, a member of a SNARE complex, which is associated with a higher risk of AD [87]. Furthermore, the FC of WD-fed pigs had increased gene expression levels of calcium sensors: calmodulin 1 (*CALM1*), which has been implicated in NDs, and synaptotagmin 2 (*SYT2*), a gene upregulated in a mouse model of AD [88,89]. Similarly, Vesicle Amine Transport 1 (*VAT1*), an integral protein of cholinergic synaptic vesicle, was upregulated in WD pigs, which is correlated to AD pathologies in patients [90].

## 5. Conclusions

In conclusion, our findings detected the molecular mechanisms by which diet-induced liver pathology leads to neurodegeneration. After 10 weeks on the Western high-fat, high-fructose diet, the frontal cortex of juvenile pigs showed dysregulation of multiple gene pathways involved in the canonical Wnt/beta-catenin signaling, cytoskeleton/ECM organization, and the mitochondrial respiratory chain. All these pathways are critical for neuronal survival and synaptic plasticity and are known to be compromised in the aging brain, Alzheimer’s disease, and other NDs. Importantly, the Western diet led to neuronal loss in the frontal cortex, yet there were no changes in the animals’ neurobehavior. Overall, this work identified strong evidence at the molecular level linking early life diet-induced MASLD and neuronal loss in the FC.

## Figures and Tables

**Table 1:** The composition of the standard diet (SD) and Western diet (WD) fed to juvenile Iberian pigs for 10 consecutive weeks. Values are expressed in percentages.

Item	SD	WD
Whey protein concentrate ^1^	8.5	8.9
Fructose ^2^	0	12
Dextrose ^2^	6	3
Fat Pak 80 ^3^	3.2	0
Hydrogenated lard ^4^	0	3.7
Hydrogenated coconut oil ^2^	0	5
Corn oil ^5^	3.2	0
Xanthan gum ^6^	0.4	0.4
Vitamin premix ^7,8^	0.32	0.32
Mineral premix ^7,8^	1.2	1.2
Cholesterol ^7^	0	0.6
Water	77.2	64.4

^1^ 80% whey protein concentrate (Hilmar Ingredients, Hilmar, CA, USA). ^2^ Tate & Lyle, Hoffman Estates, IL, USA. ^3^ Advanced Fat-Pak 80 (Milk Specialties Global Animal Nutrition, Eden Prairie, MN, USA). ^4^ Armour, Grand Prairie, TX, USA. ^5^ Healthy Brand Oil Corporation, Queens, NY, USA. ^6^ NutraBlend, Neosho, MO, USA. ^7^ Dyets Inc., Bethlehem, PA, USA. ^8^ Provided per kilogram of vitamin premix (vitamin A: 4,409,171 IU; vitamin B-12: 15.4 mg; vitamin D-3: 661,376 IU; vitamin E: 17,637 IU; menadione: 1764 mg; D-pantothenic acid: 11,023 mg; riboflavin: 3307 mg; phytase: 200,000 FTU; niacin: 19,841 mg). Provided per kilogram of mineral premix (Cu: 11,000 mg; Fe: 110,000 mg; I: 200 mg; Mn: 26,400 mg; Se: 200 mg; Zn: 110,000 mg).

**Table 2:** Daily nutrient and metabolizable energy of the standard diet (SD) and Western diet (WD) fed to juvenile Iberian pigs for 10 consecutive weeks. Values are calculated and expressed as fed. BW, body weight.

Item	SD	WD
Feed amount, L/kg BW/day	0.18	0.18
Dry matter, g/kg BW/day	40.8	62.6
Crude protein, g/kg BW/day	12.9	13
Metabolizable energy, kcal/kg BW/day	199.3	302.6
Carbohydrates, g/kg BW/day	12.8	29.1
Ether extract, g/kg BW/day	11.2	16.7

**Table 3:** Serum biochemistry and quantitative assessment of hepatic histology in juvenile Iberian pigs fed the standard (SD) and Western (WD) diets for 10 consecutive weeks.

Item ^1^	SD	WD
No. pigs (pen)	8 (4)	10 (5)
Sex (M/F)	6/2	6/4
Liver histology ^2^		
Steatosis	0.13 ^a^ ± 0.32	3.50 ^b^ ± 0.53
Ballooning	0 ± 0	0.30 ± 0.48
Mallory–Denk Bodies	0 ± 0	0.30 ± 0.48
Inflammation	1.0 ± 0	1.33 ± 0.42
Necrosis	0 ^d^ ± 0	0.70 ^e^ ± 0.58
Ki67^+^ cells ^3^	6.50 ^a^ ± 3.45	14.7 ^b^ ± 6.85
Composite lesion score	1.13 ^d^ ± 0.35	6.00 ^e^ ± 0.82
Serum biochemistry		
Alanine aminotransferase, U·L^−1^	19.5 ^a^ ± 3.5	62.2 ^b^ ± 40.4
Aspartate aminotransferase, U·L^−1^	26.9 ± 12.8	199.6 ± 149.2
Alkaline phosphatase, U·L^−1^	332.9 ± 59.8	391.6 ± 149.2
γ-glutamyl transferase, U·L^−1^	29.1 ^a^ ± 5.91	56.8 ^b^ ± 29.4
Lactate dehydrogenase, U·L^−1^	959.3 ^a^ ± 1078.3	3114.2 ^b^ ± 1492.7

^1^ Data are means ± SDs. ^2^ Steatosis: 0 (absent), 1 (<10%), 2 (10–25%), 3 (26–50%), 4 (>50%); ballooning, Mallory–Denk Bodies, fibrosis, inflammation, and necrosis: 0 (absent), 1 (minimal), 2 (mild), 3 (moderate), 4 (severe); composite lesion score: sum of all histological scores. ^3^ Ki67^+^: percentage of proliferative cells in liver. *p*-Values were calculated by one-way ANOVA and adjusted by post hoc Tukey test. Labeled means without a common letter differ: ^ab^ *p* ≤ 0.05, ^de^ *p* ≤ 0.01.

**Table 4:** Gene ontology (GO) terms up- and downregulated in the frontal cortex tissue of juvenile pigs fed the Western diet (WD) in comparison to the standard diet (SD). In silico functional enrichment analyses were conducted on differentially expressed genes (DEGs) at a 5% false discovery rate to determine the GO terms related to biological processes, cellular components, and molecular functions using the Database for Annotation, Visualization, and Integrated Discovery (DAVID) software version 6.8.

Biological Process (BP)	*p*-Value
**Upregulated WD vs. SD**	
mitochondrial respiratory chain complex I assembly	0.0000
mitochondrial electron transport, ubiquinol to cytochrome c	0.0140
vesicle-mediated transport	0.0150
**Downregulated WD vs. SD**	
cell migration	0.0012
canonical Wnt signaling pathway	0.0013
axon guidance	0.0024
signal transduction	0.0038
regulation of GTPase activity	0.0055
regulation of cell migration	0.0130
ceramide biosynthetic process	0.0150
small GTPase mediated signal transduction	0.0250
cytoskeleton organization	0.0270
extracellular matrix organization	0.0320
**Cellular Component (CC)**	*p*-value
**Upregulated WD vs. SD**	
mitochondrial respiratory chain complex I	0.0000
perikaryon	0.0011
mitochondrial inner membrane	0.0020
respiratory chain	0.0031
mitochondrion	0.0045
mitochondrial nucleoid	0.0080
voltage-gated sodium channel complex	0.0150
**Downregulated WD vs. SD**	
cell surface	0.0000
basement membrane	0.0001
focal adhesion	0.0003
beta-catenin-TCF complex	0.0004
integral component of plasma membrane	0.0075
extracellular matrix	0.0077
adherens junction	0.0230
cell junction	0.0250
**Molecular Function (MF)**	*p*-value
**Upregulated WD vs. SD**	
voltage-gated ion channel activity	0.0039
oxygen binding	0.0260
electron carrier activity	0.0300
**Downregulated WD vs. SD**	
extracellular matrix structural constituent	0.0000
GTPase activator activity	0.0001
calcium ion binding	0.0001
guanyl-nucleotide exchange factor activity	0.0002
Wnt-protein binding	0.0011
oxidoreductase activity	0.0045

## Data Availability

Data are contained within the article and additional details and materials are available upon request from the authors.
